# Calculated and measured radiation dose for the low energy xoft axxent eBT X-ray source

**DOI:** 10.1186/s13104-023-06287-1

**Published:** 2023-02-28

**Authors:** Sümeyra Can, Özge Atilla, Didem Karaçetin

**Affiliations:** Basaksehir Cam and Sakura City Hospital Radiation Oncology Department, 34480 Basaksehir Istanbul, Turkey

**Keywords:** Dose measurements, Film Dosimetry, Radiation dose, Xoft Axxent eBT

## Abstract

**Purpose:**

In this study, it was aimed to evaluate the functionality to deliver different prescription dose except 20 Gy for the Xoft Axxent Ebt (electronic Brachytherapy) system and analyzing the system in terms of radiation dosimetry in water and 0.9% isotonic Sodium Chloride (NaCl) solution.

**Materials and methods:**

In the Xoft Axxent eBT, different prescription dose in single fraction were calculated for different balloon applicator volumes based on source position and irradiation times. EBT-XD Gafchromic film was calibrated at 6MV photon energy. A balloon applicator filled with 0.9% isotonic NaCl solution was used to deliver a radiation dose of 20 Gy, 16 Gy, 10 Gy on the applicator surface. Then the balloon applicator was filled with water and the same measurements were repeated. Finally, the balloon applicator was irradiated by positioning it at different distances in the water phantom to simulate the isodose contour.

**Results:**

At the time the balloon applicator was filled with water and 0,9% NaCl solution, the difference between the planned dose and the absorbed dose was ~ 2% vs. 15% for 30 cc, ~ 5% vs. 14% for 35 cc and ~ 3,5% vs. 10% for 40 cc respectively. Finally, the absorbed dose at a distance of 1 cm from the applicator surface was measured as 9.63 Gy.

**Conclusion:**

In this study, it was showed that different prescription dose could be possible to deliver in the Xoft Axxent eBT system based on the standard plan. In addition, the absorbed dose was higher than the planned dose depending on the effective atomic number of NaCl solution comparing to water due to photoelectric effect in low energy photons. By measuring the dose distributions at different distances from the balloon applicator surface, the absorbed dose in tissue equivalent medium was determined and the isodose contours characteristics was simulated.

## Introduction

Electronic brachytherapy system (eBT) application has become very popular recently, with its use in the treatment of different tumors, including intracranial tumors. [1, 2]. The eBT system provides significant advantages in the treatment of skin tumors, intraoperative breast radiotherapy, and postoperative endometrial and cervical cancer treatment. It is also used in the treatment of cancers requiring high dose rate (HDR) after hysterectomy and/or external radiotherapy [3–6]. As it is known, boost irradiation and whole breast irradiation are accepted as standard treatment methods in addition to breast-conserving treatments such as primary tumor resection in early-stage breast cancers [7–10]. The treatment can be applied in the form of intraoperative radiotherapy (IORT), high-dose radiation can be given to the tumor bed in a single fraction, and the patient’s radiotherapy can be completed after breast-conserving surgery [11, 12]. Recently, breast IORT application with Xoft Axxent eBT system has become a common treatment method worldwide and it was first used in the boost treatment of a breast cancer patient in January 2020 in our clinic [13].

Breast IORT is performed using the Xoft Axxent eBT system utilizing a 50 kVp soft X-ray source inside a balloon applicator placed in the breast lumpectomy cavity. The source model S700 consists of a miniature (length = 10 mm, diameter = 2 mm) x-ray tube whose welding properties are characterized according to the American Association of Physicist in Medicine (AAPM) Task Group 43 (TG-43) [14–16]. Since the source has an operating voltage of 50 kV, there is no need for shielding as in the HDR brachytherapy system where Ir192 source is used [17, 18]. In addition, the system can produce air kerma up to 1400 Gy.cm^2^.h^− 1^ with a tube current of 300 µA at an operating voltage of 50 kVp, which is approximately three times larger than the value produced by the Ir192 source used in the HDR system [19–21].

As with traditional HDR brachytherapy systems, the Xoft Axxent eBT system can deliver radiation to the patient at different source positions. With the preformed standard plan for different applicator volumes and sizes, a radiation dose of 20–21 Gy is delivered in a single fraction in IORT applications. While making these calculations, the water environment is taken as a basis, as in standard brachytherapy systems, and the dose distribution is assumed to be homogeneous [22, 23]. However, the manufacturer recommends filling the applicator with 0.9% isotonic sodium chloride (NaCl) solution instead of filling the balloon applicator with water to avoid complications when the applicator rupture. Although the concentration of the solution is low, the effective atomic number of the solution (Zeff = 7.56) is greater than the effective atomic number of water (Zeff = 7.42) because it contains sodium (Na) and chlorine (Cl) ions. As it is known, the probability of photoelectric effect is dominant for photons with low energy [19]. This photoelectric effect can cause the planned dose to differ from the absorbed dose.

In previous studies, a fixed 20 Gy radiation dose and a balloon applicator filled with 0.9% isotonic NaCl solution have been used [24]. In addition, standard plans of the system provide only the dose calculated for the target volume, so the data on the absorbed dose in critical structures are very limited. As known, Xoft Axxent eBT system was designed to deliver only 20 Gy prescription dose in single fraction. On the other hand, the primary objective of this study was to show the feasibility of different prescription doses for the Xoft Axxent eBT system if needed and to analyze the absorbed dose for different prescription dose. In the next step, it was aimed to determine the difference between the planned dose and the absorbed dose when the balloon applicator was irradiated by filling 0.9% isotonic NaCl solution and water. Finally, it was aimed to measure dose values at different distances to simulate the absorbed radiation dose and dose distribution in a tissue equivalent environment.

## Materials and methods

### Film calibration

**Fig. 1:**
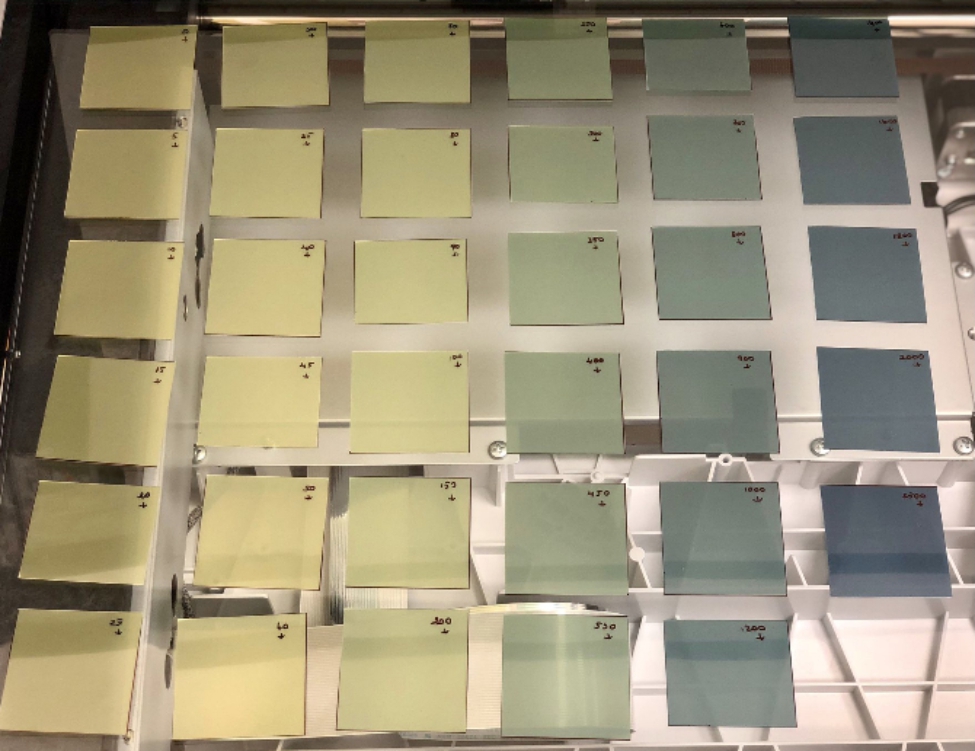
Films irradiated at different dose values for the film calibration curve

**Fig. 2:**
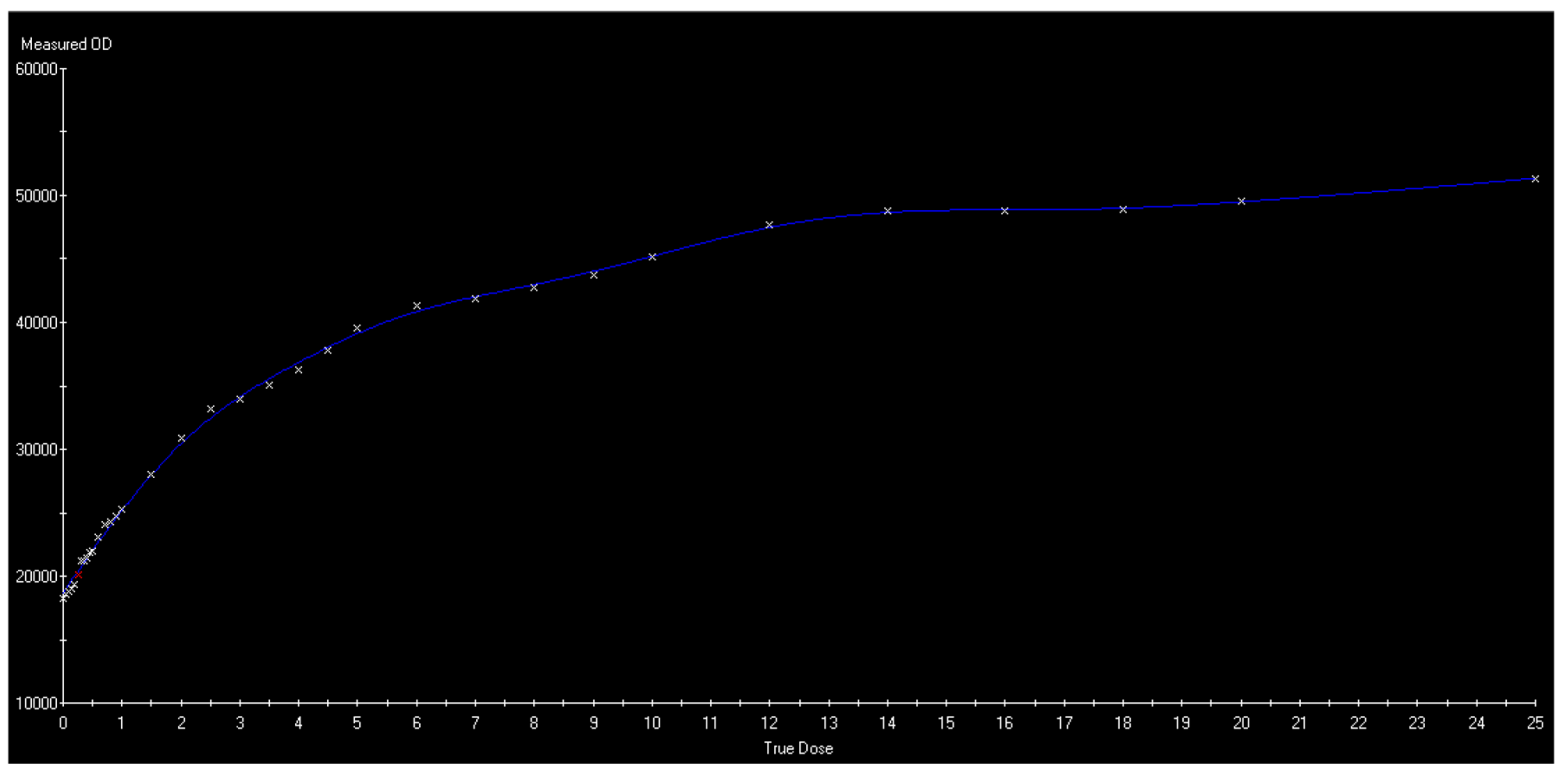
Film calibration curve

In this study, EBT-XD, a Gafchromic Film designed for the optimal dose range of 0.4 – 40 Gy, suitable for stereotactic radiosurgery (SRS) dose measurements, was used to measure the 20 Gy, 16 Gy and 10 Gy IORT dose. EBT-XD films were calibrated according to the protocol outlined in the American Association of Physicist in Medicine (AAPM) TG-55 report [25]. The films were cut to 5.0 × 5.0 cm^2^ dimensions and each film was irradiated at 6 MV photon energy produced by the Elekta Versa HDTM (Elekta, Stockholm, Sweden) linear accelerator to generate a calibration curve. Pieces of film are carefully positioned in the same direction on the central axis of a RW3 Phantom at a 10 cm depth. Dose range was from 0 to 25 Gy. After irradiation, each irradiated film was kept in the dark for 24 h. All films were then scanned with the same settings as the EPSON Expression 12000XL film scanner. During scanning, image type 48bit, 300 dpi resolution and standard scan quality were selected. Scanned films are shown in Fig. 1. Scanned films were converted to RGB colors (Red, Green, Blue) using ImageJ 1.52a program. Even though the green and the blue channels (specially the green one) provide useful information for high doses, the red component of the film was used to analyze low dose as well. Additionally, it was also used to provide standardization and consistency among low dose (< 10 Gy) and high dose (> 10 Gy) measurements. A 1.0 × 1.0 cm^2^ region of interest (ROI) was selected at the center of each film. The following equation was used to calculate the net optical density.

1 $$netOD= {OD}_{exp}- {OD}_{unexp}= {log}_{10}\frac{{I}_{unexp}}{{I}_{exp}}$$

where I_unexp_ and I_exp_ are the readings for unexposed and exposed film pieces respectively and OD was optical density of exposed and unexposed film. Afterward, a film calibration curve was obtained for different dose values and different optical densities with PTW MEPHYSTO Software for film dosimetry. The calibration curve is shown in Fig. 2.

### Different dose measurements

**Fig. 3:**
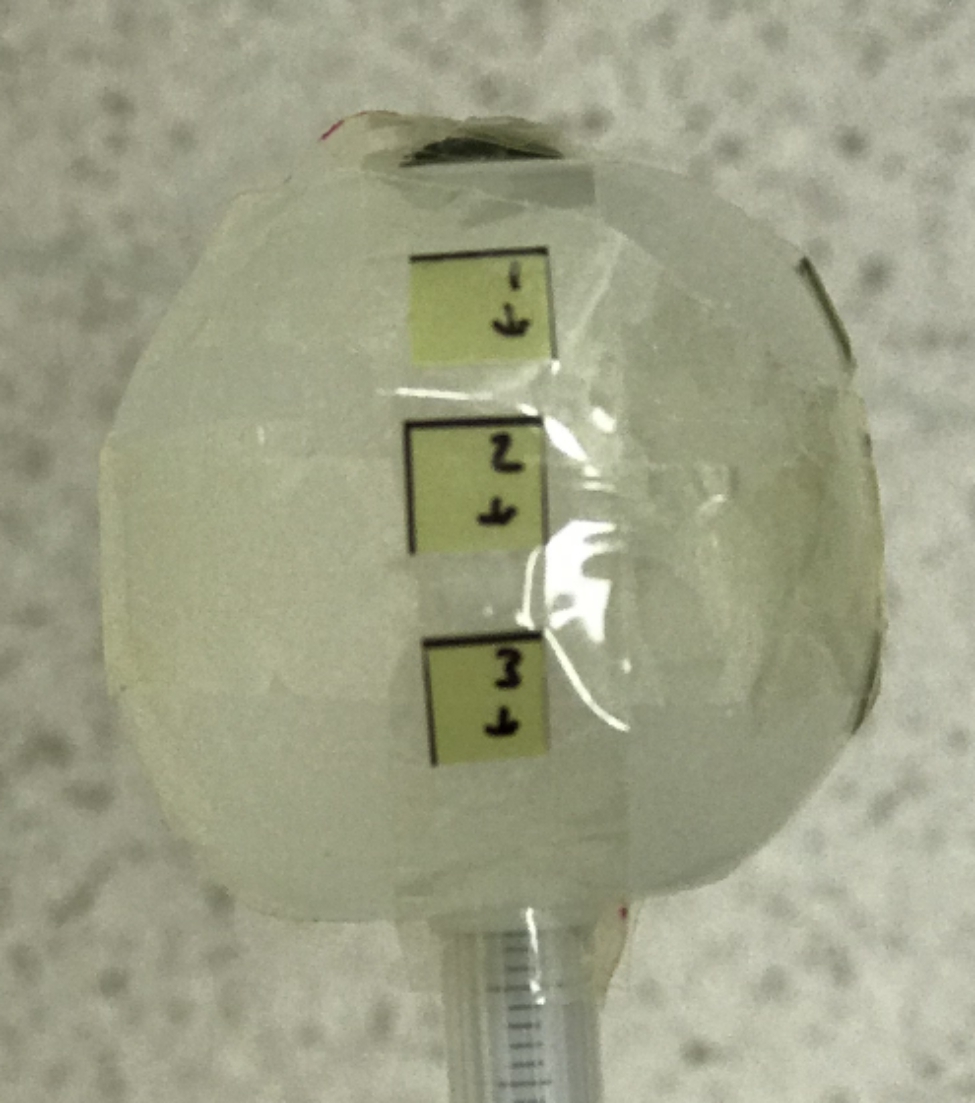
Film dosimeters placing on the balloon applicator surface

In this study, a 3–4 cm diameter balloon applicator was used for dosimetric measurements. Treatment plans were created according to the Xoft Axxent standard plan in 30 cc, 35 cc and 40 cc balloon applicator volumes to deliver 20 Gy radiation dose in a single fraction. since the radiation dose is directly proportional to the exposure time, 16 Gy and 10 Gy radiation doses were calculated for the specified balloon volumes, according to the irradiation times, provided that the standard source position remained constant. Before the measurements started, the electrometer calibration coefficient for device calibration was 0.999, the well chamber calibration coefficient was 1.050e + 11 µGy.min^− 1^.A^− 1^, the temperature was 20 ^o^C and the pressure was 755.01 mmHg. According to these values, the calibration coefficient was calculated as 0.876 and the irradiation times were calibrated according to this calibration ratio. Irradiation times calculated and calibrated according to source positions for different dose values are listed in Table 1, Table 2, Table 3. In order to measure the absorbed dose, EBT-XD Gafchromic film was cut in 7mmx7mm dimensions and numbered. the films were placed on the applicator surface from 12 different points and the measurements were performed for 20 Gy, 16 Gy and 10 Gy radiation dose on the balloon applicator surface. The films placed on the applicator surface are shown in Fig. 3. The films were kept in the dark for 24 h and scanned with the EPSON Expression 12000XL film scanner under the previously mentioned scanning conditions. The absorbed dose values in the films on the balloon applicator were analyzed according to the calibration curve.

**Table 1:** Dwell times and dwell positions calculated at different dose values for 30cc balloon applicator volume

30 cc						
	20 Gy		16 Gy		10 Gy	
Dwell Position (cm)	Calculated Time (s)	Calibrated Time (s)	Calculated Time (s)	Calibrated Time (s)	Calculated Time (s)	Calibrated Time (s)
24	13.8	12.1	11.0	9.6	6.9	6.0
23.5	186.7	163.5	149.4	130.6	93.4	81.5
23	237.1	207.7	189.7	165.8	118.6	164.6
22.5	41.5	36.4	33.2	29.0	16.6	14.5
22	39.5	34.6	31.6	27.6	15.8	13.8

**Table 2:** Dwell times and dwell positions calculated at different dose values for 35cc balloon applicator volume

35 cc						
	20 Gy		16 Gy		10 Gy	
Dwell Position (cm)	Calculated Time (s)	Calibrated Time (s)	Calculated Time (s)	Calibrated Time (s)	Calculated Time (s)	Calibrated Time (s)
24.5	7.4	6.5	5.9	5.2	3.7	3.2
24	90.6	79.1	72.5	63.4	45.3	39.4
23.5	161.2	140.7	128.9	112.7	80.6	70.1
23	184	160.6	147.2	128.7	92	80.0
22.5	163.1	142.4	130.5	114.1	81.6	71.0
22	11.1	9.7	8.9	7.8	5.6	4.9

**Table 3:** Dwell times and dwell positions calculated at different dose values for 40cc balloon applicator volume

40 cc						
	20 Gy		16 Gy		10 Gy	
Dwell Position (cm)	Calculated Time (s)	Calibrated Time (s)	Calculated Time (s)	Calibrated Time (s)	Calculated Time (s)	Calibrated Time (s)
23.5	312.4	271.2	249.9	218.7	156.2	135.5
23	297.4	258.1	237.9	208.2	148.7	129.0
22.5	51.2	44.4	40.9	35.9	25.6	22.2
22	8.3	7.2	6.7	5.8	4.2	3.6

### 0.9% Isotonic NaCl solution vs. water

According to the Xoft Axxent eBT system manufacturer’s criteria, it is recommended to fill the balloon applicator with 0.9% isotonic NaCl solution instead of water. The balloon applicator was first filled with water and irradiated to determine whether there was a difference between the planned dose and the absorbed dose. After irradiation, the applicator was emptied and filled with 0.9% isotonic NaCl solution, and the same procedures were repeated. The standard plan was created for balloon applicator volumes of 30 cc, 35 cc and 40 cc to deliver a radiation dose of 20 Gy in a single fraction. For dose measurements, EBT-XD Gafchromic film was cut to 7 mm x 7 mm and numbered. Films were placed on the applicator surface and measurements were taken from 12 different points for each balloon applicator volume. After irradiation, the films were scanned using the EPSON Expression 12000XL film scanner under the above-mentioned scanning conditions after being kept in the dark for 24 h. Dose values in the balloon applicator were obtained for 0.9% isotonic NaCl solution as well as water based on the calibration curve created earlier and uncertainty was calculated with the following Eq. 

2 $$S= \sqrt{\frac{\sum _{i=1}^{n}{({X}_{i}-\overline{X})}^{2}}{n-1}}$$

where n is the measurement points (n = 12) in the equation, x_i_ is the absorbed dose in i^th^ film and x represents the average dose absorbed in all films. An inhomogeneity correction factor was applied as the ratio of absorbed dose in heterogeneous medium to absorbed dose in homogenous medium. To evaluate the quantitative analysis of dose distribution and dose difference for both films, Gamma analysis was performed with the PTW VeriSoft v. 8.0.1 program. Since the dose distribution is based on the water environment defined by the brachytherapy dose calculation criteria, films that irradiated the balloon applicator and filled with water were considered as reference films. For gamma analysis, the distance to the agreement condition was 1 mm, 10% threshold and the criterion for dose change was 1% according to the reference dose. For regions with gamma > 1, scores corresponding to those that did not meet the acceptance criteria were considered to not meet the acceptance criteria. Thus, the accuracy of the absorbed dose in both films was quantitatively evaluated by gamma analysis.

### Dose distribution in water

**Fig. 4:**
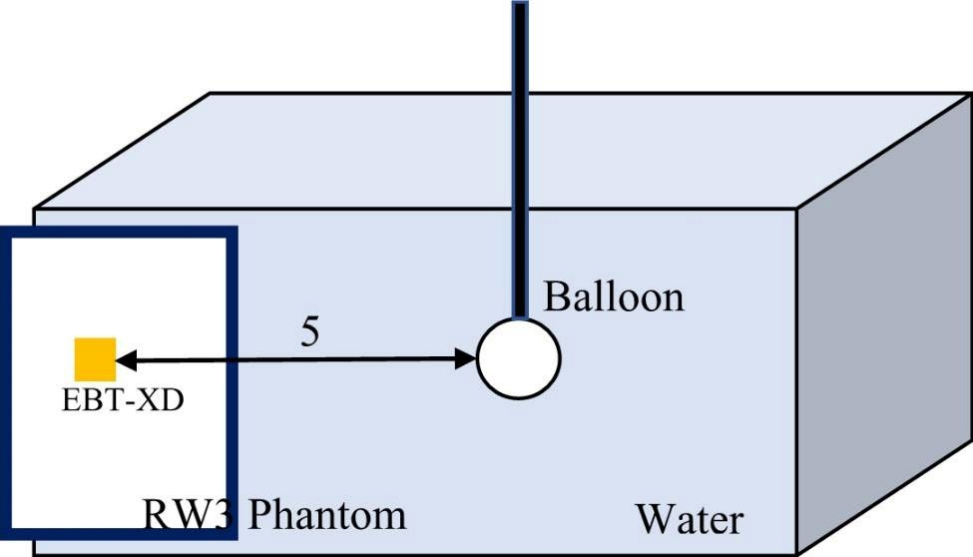
Setup position for dose distribution measurement at different distances

To simulate the dose distribution in tissue equivalent material, measurements were performed in water at 1 cm intervals starting from 1 cm from the applicator surface and up to a distance of 5 cm. EBT-XD Gafchromic film was cut to 5 cm x 5 cm dimensions and numbered to measure doses. The films were placed on the solid phantom surface so that the center of the film was aligned with the center of the balloon applicator. The set-up position for measurement in water is shown in Fig. 4. The films were irradiated according to the atlas plan created for 30 cc balloon volume. After irradiation, the films were scanned using the EPSON Expression 12000XL film scanner under the above-mentioned scanning conditions after being kept in the dark for 24 h. According to the dose values obtained for different distances, the isodose contours depending on the distance from the balloon applicator surface was simulated in the MATLAB (Version 2021a) simulation program via Simulink tool. This tool provided a virtual representation of our measurement system using mathematical relationships.

## Results

### Dosimetric analysis for different prescription dose

As known, Xoft Axxent eBT system was designed to deliver only 20 Gy prescription dose in single fraction. On the other hand, in the study, the functionality of the Xoft Axxent eBT system for different prescription doses was investigated. The 30 cc, 35 cc and 40 cc balloon volume was filled with 0.9% isotonic NaCl solution. The measurements were first made according to the standard plan which delivers 20 Gy to the applicator surface. According to the results obtained, the absorbed dose was approximately 23 Gy, 22.8 Gy and 22.1 Gy for the 30 cc, 35 cc and 40 cc balloon volumes, respectively. Accordingly, the difference between the planned dose and the absorbed dose was calculated as 15%, 14% and 11%, for the given balloon volumes. Measurements were repeated for 16 Gy and 10 Gy prescription dose under the same conditions. When a 16 Gy radiation dose was given, the absorbed dose values obtained for the 30 cc, 35 cc and 40 cc balloon volumes were 18.2 Gy, 18.3 Gy and 18.1 Gy, respectively. At a radiation dose of 10 Gy, the absorbed dose values were 10.9 Gy for 30 cc and 10.8 Gy for 35 cc. and 10.5 Gy for 40 cc. The planned dose and the absorbed dose for different prescription dose was listed in Table 4. Accordingly, it has been shown that different prescription dose can be given based on different irradiation times by using a standard plan applied to deliver 20 Gy radiation dose in the system.

**Table 4:** % difference between the planned dose and measured dose taken with 0.9% isotonic NaCl solution

	Aplicator Volume (cc)	Measured Dose (Gy)	Planned Dose vs Measured Dose (%)
**20 Gy**	**30 cc**	22.92 ± 2.11	14.60
	**35 cc**	22.88 ± 1.55	14.40
	**40 cc**	22.14 ± 1.39	10.70
**16 Gy**	**30 cc**	18.18 ± 2.77	13.62
	**35 cc**	18.32 ± 3.52	14.50
	**40 cc**	18.06 ± 3.78	12.87
**10 Gy**	**30 cc**	10.90 ± 0.88	9.00
	**35 cc**	10.79 ± 0.59	7.90
	**40 cc**	10.41 ± 0.68	4.10

### 0.9% isotonic NaCl solution vs. water

**Fig. 5:**
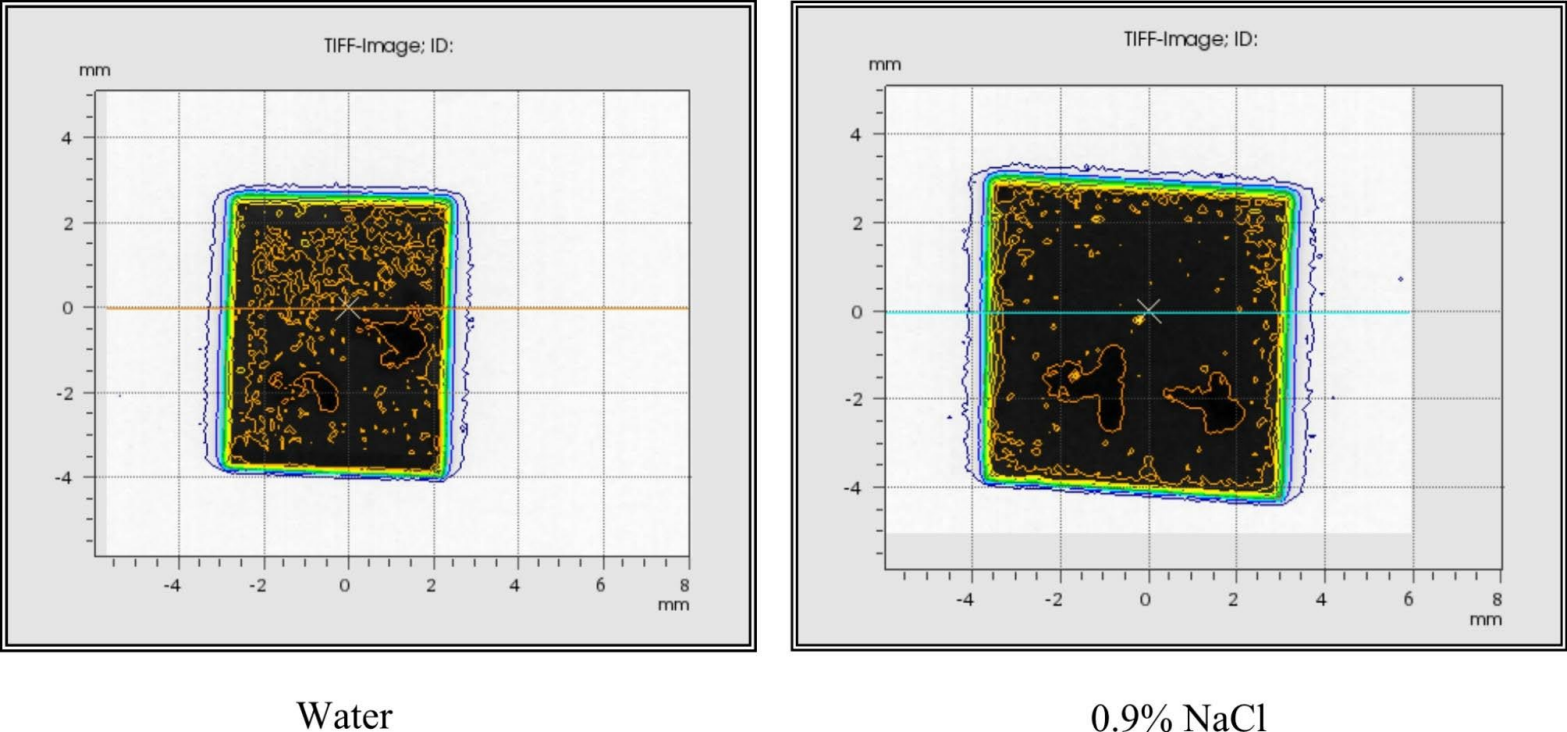
Films irradiated when the balloon applicator is filled with NaCl and water

In the Xoft Axxent eBT system, as in standard brachytherapy systems, dose calculations are made in the water environment and the dose distribution is considered to be homogeneous. However, according to the manufacturer’s criteria, it is recommended to fill the applicator with 0.9% isotonic NaCl solution instead of water to prevent complications in case of rupture of the applicator. A comparison of the absorbed dose was made for the balloon applicator filled with 0.9% isotonic NaCl solution and water. In order to make this measurement, the Xoft Axxent atlas plan was created to give 20 Gy radiation dose for 30 cc, 35 cc and 40 cc volumes. When the balloon applicator is filled with water, the absorbed dose is 20.4 Gy for the 30 cc, 20.9 Gy for the 35 cc and 20.7 Gy for the 40 cc. Provided the balloon applicator was filled with water, the difference between the planned dose and the absorbed dose was 1.9%, 4.8% and 3.5% for the 30 cc, 35 cc and 40 cc balloon applicator volumes, respectively. When the balloon applicator was filled with 0.9% isotonic NaCl solution, the absorbed dose was 23 Gy, 22.8 Gy and 22.1 Gy for 30 cc, 35 cc and 40 cc balloon volumes. Accordingly, it was observed that 0.9% isotonic NaCl solution caused an increase in the absorbed dose due to the photoelectric effect. This also caused an inhomogeneous dose distribution on the film. The values of the measurements made with 0.9% isotonic NaCl solution and water are given in Table 5. In addition, a more homogeneous dose distribution was obtained when the balloon applicator was filled with water. Irradiated films are shown in Fig. 5 for both cases. Gamma analysis was performed to evaluate the dose difference and quantitative analysis of the dose distribution for both films. Films irradiated when the balloon applicator was filled with water were considered reference films. Accordingly, gamma analysis results for both films were obtained as 91.6% for 30 cc, 88.2% for 35 cc and 90.4% for 40 cc.

**Table 5:** Measurements taken with 0.9% NaCl solution and water and % difference

	Applicator Volume (cc)	NaCl (Gy)	H_2_O (Gy)	H_2_O vs NaCl (%)
	**30 cc**	22.92 ± 2.11	20.37 ± 0.74	12.51
**20 Gy**	**35 cc**	22.88 ± 1.55	20.95 ± 1.54	9.21
	**40 cc**	22.14 ± 1.39	20.72 ± 1.26	6.85

### Dose distribution in water

**Fig. 6:**
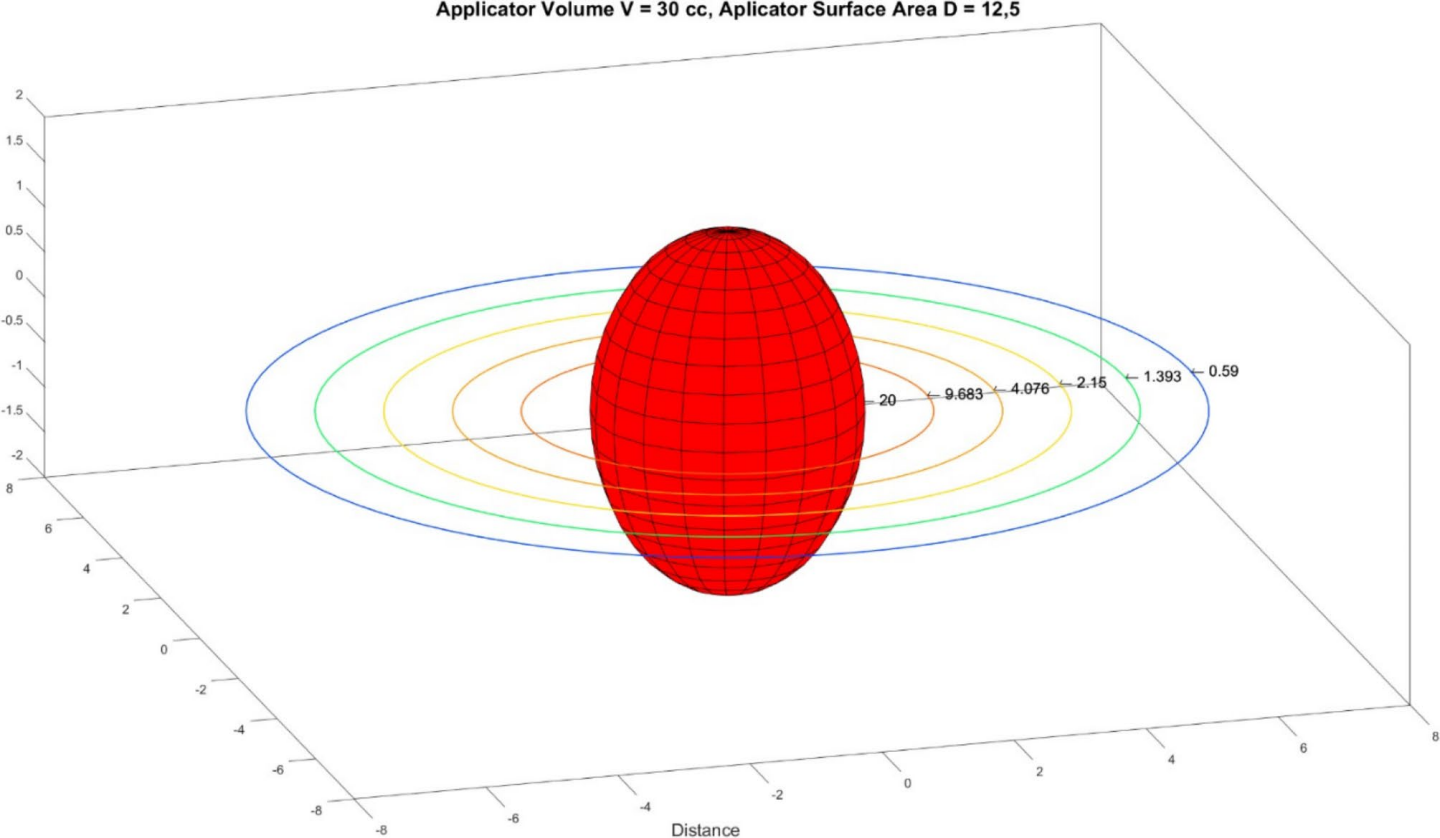
Dose Distribution and isodose contour in water for different distance from applicator surface

In the current Xoft Axxent eBT system, treatment planning only calculates the dose to be delivered to the target volume, it does not provide any data on the dose values received by the critical organs. Therefore, data on dose values of critical organs and normal tissues for IORT applications are very limited. As it is known, water is accepted as a normal tissue equivalent medium. The balloon applicator was irradiated in water at different distances to determine the dose absorbed by normal tissue. According to the manufacturer’s criteria, the dose value at a distance of 1 cm from the balloon applicator surface is stated as 10 Gy. According to the film dosimetry analysis, the absorbed dose at 1 cm from the applicator surface is 9.63 Gy, and the result is consistent with the manufacturer’s criteria. In addition, the dose absorbed at a distance of 5 cm from applicator surface was measured as 0.59 Gy. The dose values absorbed in water for different distances are given in Table 6. The MATLAB program was used to simulate the dose distribution. The dose was considered to be homogeneously dispersed in water. The simulation of the absorbed dose distribution at different distances from the applicator surface is shown in Fig. 6.

**Table 6:** Dose values measured at different distances from the balloon applicator surface

1 cm	2 cm	3 cm	4 cm	5 cm
9.68 Gy	4.07 Gy	2.15 Gy	1.39 Gy	0.59 Gy

## Discussion

Radiotherapy plays an important role in providing conservative treatment for breast cancer. Partial irradiation has recently gained more importance in breast cancers as the probability of recurrence is higher in the normal tissue close to the primary tumor [26, 27]. In this type of treatment, a high radiation dose is exposed to the tumor bed right after surgery which is called intraoperative radiotherapy. In this approach, the rate of tumor proliferation is reduced and local tumor control is achieved [28]. Even though IORT has always been taken advantage of in the treatment of early-stage breast cancer, it is recently used in boost therapy for whole breast irradiation in high risk breast cancer patients with a favorable breast cancer prognosis [29].

The Xoft Axxent eBT system is an HDR brachytherapy system using a balloon applicator and producing soft x-rays. A standard atlas plan is used to deliver a 20 Gy treatment dose in different balloon applicator sizes and volumes [30, 31]. However, the atlas plan does not provide any data on the dose received by normal tissues when calculating the amount of radiation delivered to the target. As known, the basic principle of radiotherapy is to protect the normal tissues and organs at the maximum level while delivering the treatment to the target volume. Many dosimetric analysis is developed to control the accuracy of the treatment one of which is the film dosimetry method. In The amount of absorbed dose in IORT applications was evaluated via various methods in previous studies.

Sergio Lorazes et al. evaluated the absorbed dose in a normal tissue and the prescription dose delivered to the target volume by in-vivo film dosimetry on 30 patients for breast IORT. Dosimetric measurements were performed with an XR-RW3 radiochromic film. The absorbed dose on the applicator surface was 19.8 Gy, the dose measured on the chest wall was 0.51 Gy, and the dose measured 5 cm from the applicator surface was 0.73 Gy. Based on their results, the planned dose and the absorbed dose for the target volume was almost the same, while the absorbed dose values in normal tissue were below the limit values [32].

Gage Redler et al. investigated the absorbed dose values for isotonic NaCl solution and balloon applicator filled with water in which the absorbed doses and the atlas plan dose were calculated using Monte Carlo simulation method based on the AAPM TG-43 data. For the simulation geometry, the balloon applicator was considered as a sphere and the source was considered at the center of the sphere. Absorbed dose values for a single dwell position - instead of multiple dwell positions - were calculated by the Monte Carlo method. According to the obtained results, every time the balloon applicator is filled with isotonic NaCl solution, the absorbed dose is 4-6.6% higher than the absorbed dose at the time of being filled with water [24].

Benefiting from Monte Carlo method, Mark J. Reward et al. investigated the radial dose distribution and energy spectrum of the Xoft Axxent eBT system with S700 source model for different operating voltages. They reached the conclusion that the S700 source model had similar photon penetration and percent depth dose rate with the sources emitting low energy photons [33].

On the other hand, this study analyzes the accuracy of the system for different treatment doses over different dwell times according to different standard plans of the system. According to the film dosimetry analysis, the difference between the planned dose and the absorbed dose is 2 Gy and 1 Gy, respectively whenever 16 Gy and 10 Gy treatment doses are given. Thus, the suitability of the system to apply different treatment doses based on dwell times on the standard plan was calculated. In addition, the presence of a difference in the absorbed dose values when the balloon applicator was filled with the isotonic NaCl solution against water was investigated. All measurements were performed for a multiple dwell position, not a single one. As a result, the average dose obtained on the applicator surface was 22.6 Gy when the balloon applicator was filled with isotonic NaCl solution, while the average absorbed dose on the applicator surface was 20.6 Gy when the applicator was filled with water. Accordingly, the fact that the isotonic NaCl solution caused an increase of approximately 9% in the absorbed dose compared to the water was determined. Although the concentration of the solution is low, the effective atomic number of the solution (Zeff = 7.56) is greater than the effective atomic number of water (Zeff = 7.42) because it contains sodium (Na) and chlorine (Cl) ions. As it is known, the probability of photoelectric effect is dominant for photons with low energy. It was observed that this photoelectric effect caused the planned dose to differ from the absorbed dose. Additionally, the change in absorbed dose in water at different distances was analyzed. As a result, since water is accepted as tissue equivalent in radiotherapy, the aim was to have an insight about the radiation dose absorbed in a normal tissue. According to the manufacturer’s criteria, the dose value of Xoft Axxent eBT system at a distance of 1 cm from the balloon applicator surface is 9–10 Gy in the tissue. In this study, the absorbed dose at a distance of 1 cm from the applicator surface was obtained as approximately 10 Gy in the measurements taken in water, and this result was observed to be consistent with the literature. In addition, the absorbed dose value was measured as < 1 Gy at a distance of 5 cm from the applicator surface. This results were similar to the previous study performed with EBT-XD film dosimetry during breast IORT. Accordingly, the absorbed dose value in normal tissue and critical organs was observed to be much lower than the limit values for the Xoft Axxent eBT system. Unlike the others, dosimetric measurements in this study were performed using EBT-XD gafchromic film. EBT-XD was preferred because it is close to tissue equivalent material and allows dose reading up to a maximum of 40 Gy. However, the uncertainty in the film dosimeter used was one of the limitations of this study. While the absorbed dose-energy dependence is < 1% for the EBT-XD film in the photon energy range of 100 keV – 18 MeV, the absorbed dose energy – energy dependence is > 1% when the photon energy is ≤ 100 keV. Since the photon energy in the Xoft Axxent eBT system is ≤ 50 keV, uncertainty was observed between the planned dose and the absorbed dose in the measurements taken with the EBT-XD film [32]. However, this result is within the dose uncertainty limits of ± 15% for brachytherapy according to AAPM TG-40 data [34]. Additionally, understanding the individual factors that contribute to the overall uncertainty is crucial to ensuring the accuracy of the treatment. The most important factor in our measurements is that the energy range of the film dosimetry is 10 keV – 18 MeV.

## Conclusion

According to the results obtained, the radiation dose absorbed in the normal tissue was obtained much lower than the limit values due to the soft x-ray production in the Xoft Axxent eBT system. In addition, film dosimeter analysis shows the isotonic NaCl solution causes an increase in the absorbed dose compared to the water due to the dominance of the photoelectric effect at low energies. In the system, a delivery of 20 Gy radiation dose in a single fraction and the treatment dose is calculated with the atlas plan based on different applicator sizes and volumes. However, the system’s ability to deliver different treatment doses by calculating the dose based on dwell times was shown in this study, which was obtained with the standard atlas plan.

## Limitations

There are two limitations in this study. First, dosimetric analysis was performed with EBT-XD film dosimeter which is tissue equivalent material. While the absorbed dose-energy dependence is < 1% for the EBT-XD GafChromic™ film in the photon energy range of 100 keV – 18 MeV, the absorbed dose energy – energy dependence is > 1% when the photon energy is ≤ 100 keV. Since Xoft Axxent xBT System has low energy x-ray source produced 50 kVp, absorbed dose – energy dependence was > 1% for the EBT-XD GafChromic™ film. Moreover, beam hardening effect, that occurs when an x-ray beam composed of a range of energies permeates an object and ends with specific weakening lower energy photons, was not included to calibrate EBT-XD GafChromic™ film. In this study, film calibration was performed with 6 MV photon energy to eliminate beam hardening effect, however, dosimetric measurements was done for the low energy photons.

## Data Availability

All data and materials will be provided by the corresponding authors upon request.

## Abbreviations

eBT: Electronic Brachytherapy
HDR: High Dose Rate
IORT: Intraoperative Radiation Therapy
AAPM: American Association of Physicist in Medicine
NaCl: Sodium Chloride
Z_eff_: Effective atomic number
OD: Optical Density
